# Porvac^®^ Subunit Vaccine Protects Against Three Field Isolates of Classical Swine Fever Virus

**DOI:** 10.3390/vaccines13020196

**Published:** 2025-02-17

**Authors:** Yusmel Sordo-Puga, María Pilar Rodríguez-Moltó, Danny Pérez-Pérez, Paula Naranjo-Valdés, Talía Sardina-González, Mary Karla Méndez-Orta, Elaine Santana-Rodríguez, Milagros Vargas-Hernández, Carmen Laura Perera, Carlos A. Duarte, Marisela Suárez-Pedroso

**Affiliations:** 1 Departamento de Salud Animal, Centro de Ingeniería Genética y Biotecnología, Apdo 6162, La Habana 10600, Cuba; pilar.rodriguez@cigb.edu.cu (M.P.R.-M.); danny.perez@cigb.edu.cu (D.P.-P.); talia.sardina@cigb.edu.cu (T.S.-G.); mary.mendez@cigb.edu.cu (M.K.M.-O.); elaine.santana@cigb.edu.cu (E.S.-R.); carlos.duarte@cigb.edu.cu (C.A.D.); marisela.suarez@cigb.edu.cu (M.S.-P.); 2 Unidad del Laboratorio Central para la Salud Animal (ULCSA), La Habana 11400, Cuba; labconestatal@hab.minag.cu; 3 Centro Nacional de Sanidad Agropecuaria (CENSA), San José de las Lajas 32700, Cuba

**Keywords:** classical swine fever, vaccine, Porvac^®^, field isolates, viral challenge

## Abstract

The control of classical swine fever (CSF) in endemic areas has been attempted with modified live vaccines. However, in some regions, the implementation of imperfect vaccination programs has led to a reduction in the genetic diversity of the circulating CSF virus (CSFV) strains and a change in their virulence. Porvac^®^ subunit vaccine has been shown to provide a rapid onset of protection against the “Margarita” strain. The aim of this study was to evaluate whether the immune response induced by Porvac^®^ is also effective against autochthonous CSFV isolates of low, medium or high virulence. All pigs vaccinated with Porvac^®^ were protected against the disease after challenge. PR-11/10–3 isolate caused a very mild disease in controls, whilst Holguin_2009 isolate produced mild to moderate signs of CSF and one of the pigs died. Finally, controls inoculated with PR-2016 isolate developed moderate to severe signs of CSF and two of them died. Viral replication was detected in controls, but not in pigs immunized with Porvac^®^. Finally, anti-E^rns^ antibodies were induced in five out of six control pigs but not in any of the vaccinated pigs. These results support the use of Porvac^®^ for the control and elimination of CSF in Cuba and other endemic regions.

## 1. Introduction

Classical swine fever (CSF) is a highly contagious disease that causes significant losses in the pig industry worldwide, with developing countries particularly affected [1,2]. The etiological agent is the CSF virus (CSFV), an RNA envelope virus from the *Flaviviridae* family. CSF is enzootic in areas of Central America and the Caribbean, South America, Southeast Asia, and Eastern Europe. The control of the disease in endemic areas has been attempted through the application of modified live vaccines (MLV). However, the implementation of prolonged and imperfect vaccination programs has led to a reduction in the genetic diversity of the circulating strains of CSFV and to a change in the pathogenicity of novel, less virulent and more persistent virus strains [3,4,5,6].

In Cuba, all pigs have been vaccinated with a lapinized MLV (Labiofam C strain) since 1965. For decades, the country remained CSF-free, but a viral outbreak was detected in 1993 [7]. Molecular epidemiology studies suggested that the reemergence of the disease was related to the escape of the highly virulent “Margarita”, a 1958 isolate used in challenge experiments for vaccine lot release [8].

Ten years after this reemergence, the disease had become endemic in the country. The high rate of non-synonymous mutations found in the fragment of E2 sequence, in conjunction with the tendency towards less virulent forms of the disease, suggested that the evolution of the virus had been driven by the selective pressure associated with a prolonged and inefficient vaccination program [9]. Subsequently, it was demonstrated that the inability of the MLV in use to confer sterilizing immunity resulted in a bottleneck effect that facilitated the emergence of a viral population with new characteristics, lower virulence and pathogenicity as possible escape mutants to the neutralizing antibodies (NAb) elicited by this vaccine [5]. These authors identified six non-synonymous mutations, which were located mainly in the B/C domain, towards the N-terminus of the E2 protein. This domain has been identified by other researchers as the most variable within the protein and where mutations that allow the virus to escape from NAb are selected [10,11].

Subunit vaccines are now available which, in contrast to MLV, induce antibodies that can be theoretically distinguished from those generated by the natural virus (DIVA). The subunit vaccine Porvac^®^ was developed as an alternative to traditional MLV in endemic areas such as Cuba. The active ingredient of Porvac^®^ is the chimeric protein E2-CD154, formed by the external region of the E2 glycoprotein of the CSFV, coupled to the extracellular segment of the pig CD154 molecule. Porvac^®^ provides a very rapid onset of protection, similar to that of MLV [12].

Nevertheless, the highly virulent ancestral “Margarita” strain of CSFV has been exclusively used in all challenge experiments conducted in Porvac^®^-vaccinated pigs. It is important to highlight that the sequence of the E2 protein in the “Margarita” strain is identical to the E2 included in Porvac^®^. The efficacy of Porvac^®^ against other viral strains that are not identical to the vaccinal antigen has yet to be demonstrated.

Given that Porvac^®^ is being used as part of the control and elimination program for CSFV in Cuba, it is of great relevance to demonstrate its efficacy in a controlled confrontation experiment against viral isolates genetically closer to the contemporary viruses circulating in our country. Many of these circulating isolates are of low or medium virulence and could have evolved to escape the selective pressure exerted by the MLV [5]. The objective of this study was to evaluate whether the immunity conferred by Porvac^®^ is able to protect pigs from challenge with three autochthonous strains of CSFV of low, medium or medium/high virulence, isolated in different regions of the country between 2009 and 2016. The three selected isolates exhibit a number of amino acid changes in the E2 protein relative to the ancestral “Margarita” strain and are more representative of the viruses that are currently circulating in the field.

## 2. Materials and Methods

### 2.1. Viruses and Cells

The three Cuban field CSFV strains used in the challenge experiments were isolated from pigs with mild clinical signs, mainly respiratory distress, by the Virology Laboratory of the National Center for Animal Health (CENSA, Mayabeque, Cuba). Holguin_2009 (GenBank accession number HE584533) was isolated from Holguín province in 2009 and partially sequenced [5]. Subsequently, it was identified as a medium-virulence strain [13]. PR-11/10-3 (GenBank accession number KX576461) was isolated and partially sequenced from the western Pinar del Río province [5], and its genome was later completely sequenced [14]. It is also referred to as CSF1058 in accordance with the nomenclature of the European Union Reference Laboratory for Classical Swine Fever (EURL-CSF), Hannover, Germany. It was characterized as a low-virulence isolate [13]. PR-2016 (GenBank accession number LT985811) was isolated from piglets without clinical signs of the disease in Pinar del Río province in 2016. The sequence of the E2 gene has been completely dilucidated [15]. Prior to this study, the degree of virulence of this strain had not been investigated.

The three viruses were titrated by end point dilution in PK-15 cells (ATCC CCL-33) using a peroxidase immunostaining assay with the anti-E2 monoclonal antibody (Mab) CBSSE2.3 (Center for Genetic Engineering of Sancti Spíritus (CIGB-SS), Sancti Spíritus, Cuba) conjugated to horseradish peroxidase.

The pig kidney PK-15 cell line was also used in the neutralizing peroxidase linked assay (NPLA) and virus isolation assays. PK-15 cells were grown in Dulbecco Modified Eagle Medium (DMEN) supplemented with 10% fetal calf serum (Capricorn, Burswood, Australia) and antibiotics 10,000 U/mL of penicillin/10 mg/mL streptomycin (Sigma, St. Louis, MO, USA).

### 2.2. Porvac^®^ Vaccine

Porvac^®^ subunit vaccine was kindly provided by the Center for Genetic Engineering and Biotechnology of Camagüey (Camagüey, Cuba). The active principle of this vaccine is the chimeric protein E2-CD154, formed by the fusion of the extracellular region of the E2 glycoprotein of the CSFV “Margarita” strain and the extracellular segment of the swine CD154 molecule. A lentivirus-based gene delivery system was used to generate a stable recombinant HEK 293 cell line (ATCC CRL1573) for the expression E2-CD154, as previously described [16]. E2-CD154 protein was formulated in Montanide^TM^ ISA50 V2 (SEPPIC, La Garenne-Colombes, France) using a 60/40 proportion of aqueous/oil phase and a SD-41 homogenizer (IKA, Königswinter, Germany) under Good Manufacturing Practice (GMP) conditions. The concentration of E2-CD154 in the final emulsion was 25 μg/mL. 

### 2.3. Experimental Animals

The studies were conducted under high-containment conditions in accordance with the animal welfare regulations and standards of EU Directive 2010/63/EU (EU Directive 2010/63, Official Journal of the EU) and Good Clinical Practice [17]. Crossbred Duroc/Yorkshire pigs (25–30 kg) were obtained from a CSF-free, unvaccinated herd at the National Center for Animal Production (CENPALAB, La Habana, Cuba). CENPALAB is the Cuban institution specialized in the production of quality laboratory animals. For decades, this center has maintained a CSFV-free non-vaccinated herd, which has been certified by the national veterinary authorities.

The twenty-four nine-week-old pigs included in the study received 2 kg/animal/day of food and water ad libitum. The pigs in the paddocks were tagged with visible identification marks (notches).

### 2.4. Experimental Design, Immunization Schedule and Viral Challenge

**Figure 1:**
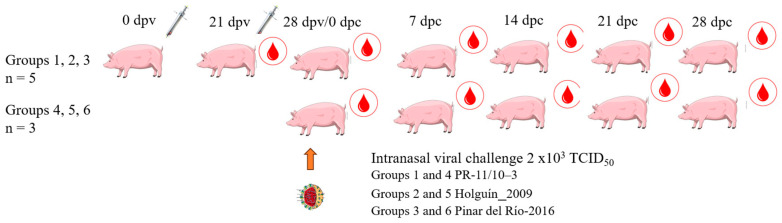
Immunization schedule and virus challenge. Groups 1–3: 5 animals vaccinated with Porvac^®^; groups 4–6: 3 non-vaccinated controls. Dpv: days post-vaccination, dpc: days post-challenge. The syringes indicate the days of vaccination. The red drops indicate the days of blood extraction. All pigs were challenged on 28 dpv/0 dpc as indicated by the orange arrow and the virus. Groups 1 and 4: challenged with isolate PR-11/10-3; groups 2 and 5: challenged with isolate Holguín_2009; groups 3 and 6: challenged with isolate PR-2016.

The animals were randomly allocated into six experimental groups (Figure 1):

The pigs in groups 1, 2, and 3 were immunized twice by intramuscular injection with the standard dose of the vaccine (2 mL of the emulsion containing 50 µg of E2-CD154 protein). The first injection was on the right side of the neck, and the second, 21 days later, on the left side. The injections were performed using 18 G X 1-inch needles in accordance with good veterinary clinical practices. The control animals (groups 4, 5, and 6) received a mock emulsion comprising Montanide™ ISA50 V2 and phosphate buffer saline.

Seven days after the second injection, all pigs were intranasally challenged with 2 × 10^3^ TCID_50_ of isolates PR-11/10-3 (groups 1 and 4), Holguín_2009 (groups 2 and 5) and PR-2016 (groups 3 and 6). Following viral challenge, the pigs were monitored daily and a clinical score was established according to the methodology established by Mittelholzer et al. [18], with some modifications. These changes consist of the exclusion of three parameters (breathing, body shape, and body tension), and the inclusion of the body temperature. Heparinized blood and serum samples were taken immediately before challenge and at 7, 14, and 21 days post-challenge (dpc). Blood was extracted via ophthalmic venous sinus puncture and collected in sterile tubes with and without an anticoagulant (VACUATTE^®^ Greiner bio-one, Frickenhausen, Germany). The blood was incubated at room temperature for two hours and then kept overnight at a temperature range of 2–8 °C before extraction, with some modifications as described elsewhere.

The study was approved and supervised by the Internal Committee for the Care and Use of Laboratory Animals (CICUAL) of CENPALAB.

### 2.5. Neutralizing Peroxidase Linked Assay (NPLA)

The serum samples were screened for the ability to neutralize the cell-culture adapted “Margarita” CSFV strain in the NPLA, following the recommendations of WOAH [19]. This assay has been approved for its routine application to Porvac^®^ batch release.

### 2.6. Detection of Antibodies Directed Against the E^rns^ Protein of CSFV

The commercial ELISA test PrioCHECK^®^ CSFV E^rns^ (Applied Biosystems, Waltham, MA, USA) was used to detect the presence of antibodies against E^rns^ protein of CSFV in the sera of the piglets after viral challenge. This is a competition ELISA, based on blocking the binding of E^rns^ MAbs to the E^rns^ protein. The assay was conducted according to the manufacturer’s instructions. A sample was regarded as positive for E^rns^ antibodies if the percent of inhibition was greater than 40%.

### 2.7. Viral Isolation

Viral isolation was performed using blood samples treated with heparin, collected at 3, 7, 14, 21, and 28 dpc. The protocol described in the Manual of World Organization for Animal Health was followed [19]. Briefly, at 28 dpc, spleens and tonsils were collected and approximately 1 cm^2^ of each tissue was macerated in 1 mL of DMEM (Sigma, St. Louis, MO, USA) supplemented with streptomycin (100 μg), 5% fetal calf serum, and penicillin (100 IU). The homogenates were suspended in 4 mL of DMEM, incubated for 1 h at room temperature, and then centrifuged for 15 min at 400 g. The resulting supernatant was transferred and preserved at −80 °C in cryovials (Sigma-Aldrich, USA). Viral isolation was conducted in PK-15 cells through two serial passages in 48-well microplates and a third passage onto 96-well microplates. Six replicates were utilized for each sample. The presence of replicating virus was detected with the monoclonal antibody CBSSE2.3 (CIGB-SS, Sancti Spíritus, Cuba) conjugated to horseradish peroxidase. The reaction was revealed with 3-amino-9-ethyl carbazole (AEC) and hydrogen peroxide. A control curve with the “Margarita” strain of CSFF with 1000, 100, 10 and 1 TCID_50_ diluted in serum was run in every assay. For the assay to be valid, it had to detect at least 10 TCID_50_ of this virus.

### 2.8. Statistical Analysis 

The normality of the data was evaluated through the application of the Kolmogorov–Smirnov and D’Agostino–Pearson tests. The Kruskal–Wallis test was employed to assess the differences in titers among more than two groups of animals, while Dunn’s multiple comparisons test was used as a post hoc test. The Mann–Whitney test was applied to compare antibody titers between the two experimental groups. All of the aforementioned analyses were conducted using the GraphPad Prism 6 statistical package (GraphPad Prism for Windows, Version 6.01, GraphPad Software, Inc., La Jolla, CA, USA). The threshold for statistical significance was set at *p* < 0.05.

## 3. Results

### 3.1. Neutralizing Antibody Response

**Figure 2:**
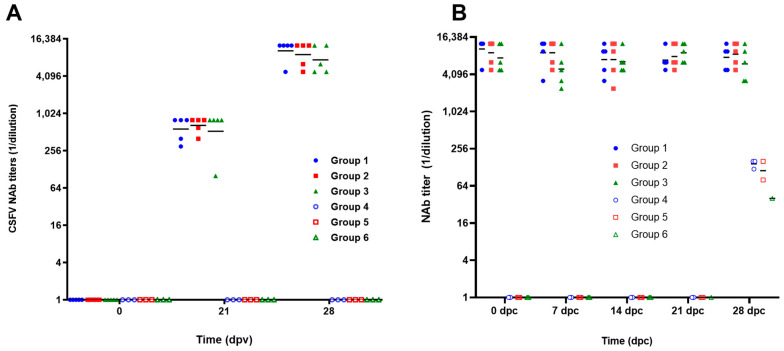
Classical swine fever virus neutralizing antibody titers. (**A**) NAb titers after two immunizations with Porvac^®^. (**B**) NAb titers after challenge with field isolates. Dpv: days post-vaccination, dpc: days post-challenge. Groups 1, 2, and 3 were immunized with Porvac^®^ on days 0 and 21; groups 4, 5 and 6 were non-immunized controls. X axis is in log2 scale. The challenge day (0 dpc) corresponds with 7 days after the second administration of the vaccine.

All animals vaccinated with Porvac^®^ developed NAb titers after the first administration of Porvac^®^. The geometric mean of the titers was 1:584, with 95% confidence intervals of 1:421 and 1:808. NAb titers were significantly increased after the booster, with a geometric mean of 1:8984 and 95% confidence intervals of 1:6874 and 1:11,574 (Figure 2A) (Mann–Whitney test, *p* < 0.0001). No significant differences in the NAb among groups 1, 2 and 3 were found after one or two administrations of Porvac^®^ (Kruskal–Wallis test, *p* > 0.05).

Seven days after the second immunization, the vaccinated animals and their controls were challenged with the three field isolates. During the four weeks following viral challenge, NAb titers in the vaccinated animals remained stable, with geometric means per group fluctuating between 1:4996 and 1:9158 (Figure 2B). No significant differences were found between the three groups vaccinated with Porvac^®^ for each of the times sampled (Kruskal–Wallis, *p* > 0.05). The animals in the three non-vaccinated groups that survived the challenge (three pigs in group 4, two in group 5 and one in group 6) developed very low NAb titers 28 days after challenge, with geometric mean of 1:108 and 95% confidence intervals of 60.11 and 193.5.

### 3.2. Body Weight After CSF Challenge

**Figure 3:**
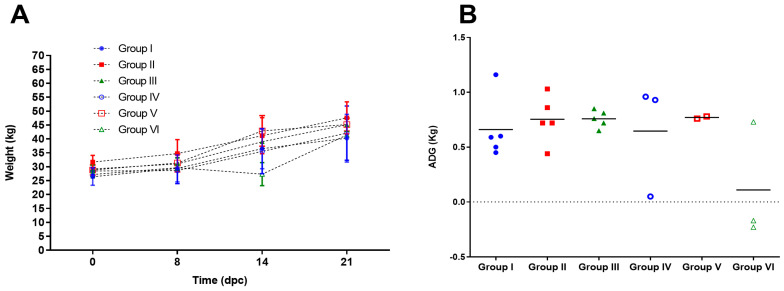
Weight of the animals after challenge. (**A**) Time course of body weight per group. Values represent the average weight at each dpc and the standard deviation of the mean. (**B**) Average daily gain (ADG) per group during 14 dpc. Values represent individual values of ADG in the period studied (from 0 to 21 dpc); horizontal lines represent the mean ADG for each group.

The vaccinated animals showed stable growth dynamics after challenge with the three field isolates (Figure 3A). The mean values in average daily gain (ADG) expressed in kg/day were 0.72 ± 0.31, 0.67 ± 0.33, and 0.70 ± 0.25 for groups 1, 2, and 3, respectively, during the 14-day period after challenge, when most of the pigs were still alive (Figure 3B).

The unvaccinated control animals challenged with PR-11/10–3 and Holguin_2009 isolates showed mean ADG values of 0.50 ± 0.63 and 0.66 ± 0.59, respectively. These values did not show statistical differences with respect to Porvac^®^ vaccinated animals challenged with the same isolates (Mann–Whitney test, *p* > 0.05). However, one out of three animals in these two groups did not have a positive ADG in that period (Figure 3B).

On the contrary, non-vaccinated control animals challenged with the field isolate PR-2016 showed a statistically significant decrease in ADG values (0.009 ± 0.36) when compared to vaccinated animals challenged with the same isolate (Mann–Whitney test, *p* < 0.05).

### 3.3. Clinical Evaluation After Challenge

#### 3.3.1. Temperature

**Figure 4:**
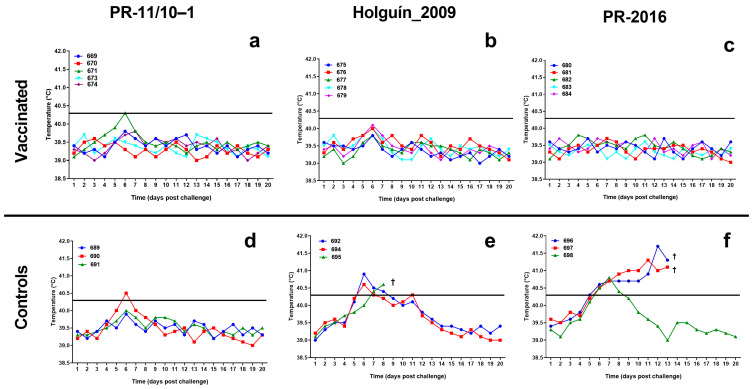
Rectal temperature of the animals after viral challenge with classical swine fever virus field isolates. (**a**) vaccinated pigs challenged with PR-11/10-1 isolate. (**b**) vaccinated pigs challenged with Holguin_2009 isolate. (**c**) vaccinated pigs challenged with PR-2016 isolate. (**d**) control pigs challenged with PR-11/10-1 isolate. (**e**) control pigs challenged with Holguín_2009 isolate. (**f**) control pigs challenged with PR-2016 isolate. Horizontal lines indicate the threshold of fever (40.3 °C). A cross (†) indicates the death of the animal.

All pigs vaccinated with Porvac^®^ maintained stable body temperatures after challenge with the three field isolates. The only exception was animal 672, which, challenged with PR-11/10–3 isolate, had a transient burst of fever on day 6, but this immediately disappeared at day 7 and the animal’s temperature remained normal for the rest of the study (Figure 4).

A similar behavior was observed in the non-vaccinated control animals challenged with PR-11/10–3 field isolate. Only one animal (690) developed rectal temperature values of 40.5 °C on the sixth day post-confrontation, but these values normalized in the following days.

All three pigs in the control group challenged with the Holguin_2009 isolate experienced fevers. Two of them had a fever from the sixth day, although the temperature normalized by 8 and 9 dpc. The third animal in this group had a sustained fever from 7 dpc until its death two days later.

The three control animals challenged with PR-2016 isolate had fevers which started 6 dpc. One of them normalized its temperature values by 9 dpc, but the other two had sustained fevers until their deaths at 13 dpc.

#### 3.3.2. CSF Clinical Score

**Figure 5:**
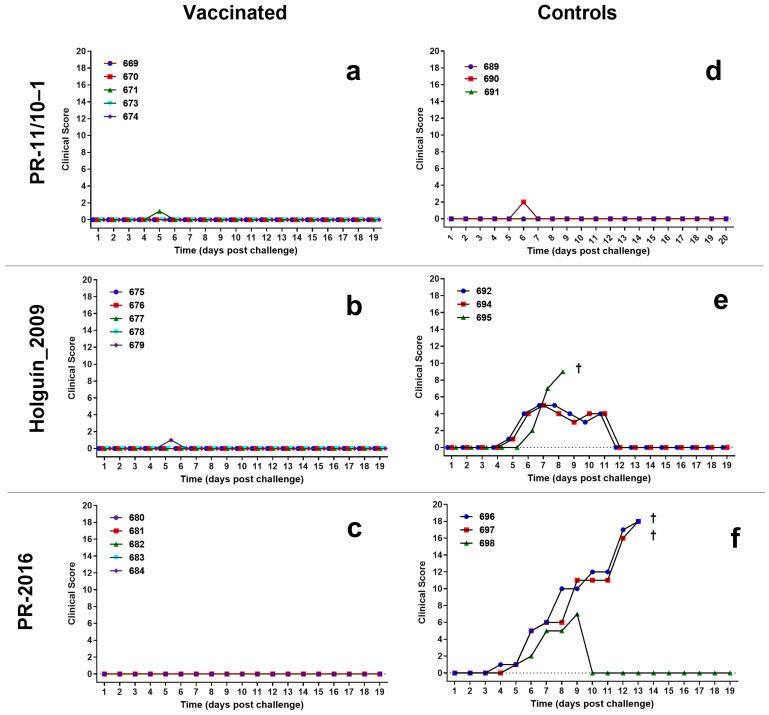
Clinical score of the animals challenged with the three field isolates of classical swine fever virus. Clinical score was compiled according to Mittelholzer et al. (**a**) vaccinated pigs challenged with PR-11/10-1 isolate. (**b**) vaccinated pigs challenged with Holguin_2009 isolate. (**c**) vaccinated pigs challenged with PR-2016 isolate. (**d**) control pigs challenged with PR-11/10-1 isolate. (**e**) control pigs challenged with Holguin_2009 isolate. (**f**) control pigs challenged with PR-2016 isolate. A cross (†) indicates the death of the animal.

Porvac^®^-vaccinated swine did not show clinical signs of CSF after challenge with the three field isolates (Figure 5). Likewise, control animals challenged with Pinar del Rio isolate did not experience clinical signs of CSF after challenge, other than a very mild reduction of vitality. None of these animals showed macroscopic pathology lesions at the moment of sacrifice. 

Two of the control animals challenged with Holguín_2009 isolate showed a transient increase in the CSFV clinical score, but all signs disappeared after 12 dpc. These two animals were also negative for macroscopic lesions at sacrifice. The third animal died suddenly at nine dpc with a drastic reduction of vitality, anorexia, and diarrhea. The macroscopic pathology analysis of this pig could not be conducted.

Finally, two non-vaccinated controls challenged with PR-2016 isolate experienced increasing clinical signs of CSF and were euthanized at 13 dpc with a drastic reduction of vitality, anorexia, diarrhea, and nervous disorders. Macroscopic pathology analysis of animal 696 showed swollen, edematous, hemorrhagic lymph nodes; marginal hemorrhages in the spleen; and pneumonia. Animal 697 exhibited ascites and pneumonia. The third animal also showed clinical signs of CSF but these had resolved completely by 10 dpc and no macroscopic pathology findings were detected at necropsy.

### 3.4. Anti-E^rns^ Antibodies

The E^rns^ antigen is not present in the Porvac^®^ vaccine, so the presence of antibodies against it is a marker of viral infection, and allows differentiation between vaccinated and infected animals. As shown in Table 1, anti-E^rns^ antibodies were detected in most of the unvaccinated control animals, with the exception of pig 689, which was challenged with PR-11/10–1 isolate. In contrast, all Porvac^®^-vaccinated animals were negative for anti-E^rns^ antibodies (Table 1). 

### 3.5. Viral Isolation

Virological analysis of the samples (blood, tonsils, spleen, and lung) showed absence of virus in all pigs vaccinated with Porvac^®^ and challenged with the different Cuban isolates (Table 2). 

In contrast, non-vaccinated control animals challenged with PR-11/10-3 (group 4) and Holguin_2009 (group 5) were positive for virus isolation from the lungs at the time of sacrifice. The exception was pig 692, which was negative at sacrifice, although it had been positive at 7 dpc in blood. In the case of the non-vaccinated animals challenged with isolate PR-2016 (group 6), viral replication could be detected in all the samples studied, with the exception of the tonsils in animal 698.

## 4. Discussion

Between 2001 and 2003, a decade after the onset of the CSF epizootic in Cuba, several strains were isolated from animals exhibiting chronic forms of the disease. These isolates showed non-synonymous nucleotide mutations in the same fragment of the partial E2 gene sequence when compared to the ancestral strain “Margarita” [9]. The results of this and other studies suggested that the evolution of CSFV in Cuba could have been driven by a positive selection pressure associated with the implementation of suboptimal vaccination programs using the lapinized MLV [9].

The lower virulence of the circulating viruses results in a mild, often undetectable clinical picture, although they can cause deficiencies in the growth of the animals and predisposition to other diseases due to the immunosuppression associated with CSFV. In endemic areas, where infected animals do not show clinical signs indicative of CSFV infection, they can maintain low viral loads for long periods of time. This facilitates viral circulation in the field and increases the likelihood that new viral variants will be selected as a result of immune system pressure over a prolonged period [13].

Porvac^®^ is a subunit vaccine whose active ingredient is the E2 protein fused to the pig CD154 antigen. The E2 gene of the “Margarita” strain was used for the construction of the HEK293 cell line expressing E2-CD154. The prevailing hypothesis regarding the origin of the CSF epizootic in Cuba, supported by the results of molecular epidemiology, is that the “Margarita” strain used in the MLV challenge tests escaped to the field [8]. Consequently, the sequence of the vaccine E2 antigen is very similar to that of the circulating viruses.

The aim of this study was to test whether pigs immunized with Porvac^®^ resisted challenge with three Cuban isolates exhibiting low or medium virulence, and which may have evolved to evade the immune response induced by the lapinized MLV. 

**Figure 6:**
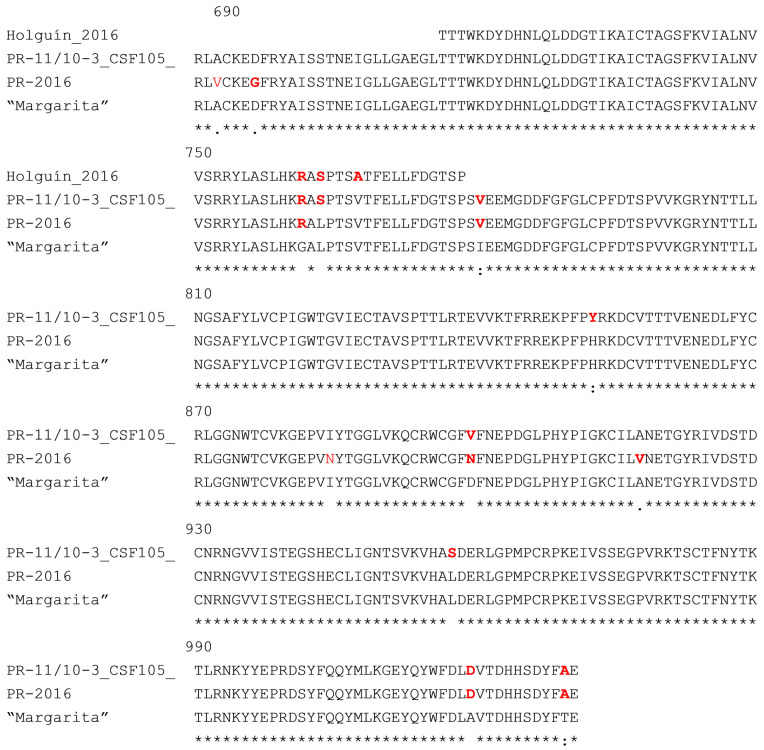
Alignment of the E2 sequences of the three Cuban isolates and the “Margarita” strain (same sequence as the E2-CD154 antigen in Porvac^®^). Differences from the ancestral “Margarita” strain are depicted in red. The numbers indicate the position of the amino acids in the CFSV polyprotein. Asterisks indicate conserved positions among the four viral strains. The sequence corresponding to the C terminal transmembrane region is not shown since this part is not included in E2-CD154.

Figure 6 shows the sequence alignment of the field isolates studied in this work and the “Margarita” strain. Only the E2 protein fragment included in the vaccine antigen E2-CD154, which does not include the transmembrane region, is shown.

The PR-11/10-3 isolate [5,14] shows nine amino acid differences from the “Margarita” strain within this region. Of these, three (G761R, L763S and I780V) are located within the B/C domain, which is the main target of neutralizing antibodies on the E2 protein [10,11]. The first of these sites responds to a selective pressure according to previous analysis performed with several Cuban CSF isolates. Notably, this change has been fixed in the population from 2001 to 2011, suggesting that it represents an important adaptive advantage for the virus [5]. This isolate is characterized by presenting the insertion of a polyuridine track in the 3′URL of the virus, which has been associated with its low virulence [14]. The Holguín_2009 isolate has three amino acid changes when compared to the “Margarita” strain in the 66 amino acid region of the E2, which has been sequenced [5]. The first two changes are identical to those observed in the isolate PR-11/10-3 (G761R, L763S), while the third is the conservative substitution V767A. All these changes are located within the B/C domain. The complete sequence of the E2 protein from isolate PR-2016 is known. This strain presents seven amino acid changes in relation to the ancestral strain “Margarita”, and two of these substitutions (G761R and D884N) have been identified as selective pressure points according to the analysis performed by Coronado et al., [15]. The first of these changes, within the B/C domain, also appears in the two other isolates. Finally, the conservative I780V substitution within the B/C domain is common to isolate PR-11/10-3.

Clinical outcome after infection with these strains in unvaccinated control animals confirmed previous results by Coronado et al. [13], who reported the low virulence of isolate PR-11/10-3, as well as the moderate virulence of isolate Holguin_2009. In the present study, control pigs infected with PR-11/10-3 did not manifest clinical signs of CSF at any time, with the exception of transient fever in one animal. However, the positive virus isolation from the lungs of the three pigs at the time of sacrifice demonstrated that the infection was effective. The virulence of the Holguín_2019 strain in this study can be described as moderate. While all three animals showed clinical signs of CSF, two of them spontaneously resolved the disease and remained asymptomatic for the remainder of the study. The third animal, however, died at 9 dpc. The virus was successfully isolated from the two surviving pigs. Prior to this study, the virulence of the PR-2016 isolate was unknown. The results obtained in this experiment suggest that this isolate is of moderate to high virulence as all three controls manifested clinical signs of CSF and two of them died at 13 dpc. However, further experiments are required for an accurate characterization of its virulence. 

In this study, a dose of 2 × 10^3^ TCID_50_ was administered via the intranasal route for the viral challenge. This dose is lower than that recommended by WOAH for challenge testing of CSF vaccines. A higher dose of virus in the challenge could result in a more acute clinical picture than that observed here. However, the intranasal infection of five-day-old pigs with 2.5 × 10^4^ TCID_50_ of the PR-11/10-3 did not produce clinical signs of CSF in a previous study [20]. Although tonsils are considered the main center for viral replication, our previous experience with low-virulence isolates suggests that they are more readily isolated from the lungs. This observation was confirmed here for the pigs challenged with isolates PR-11/10-3 and Holguín_2016, where it was possible to isolate the virus mostly from the lungs and not from other target organs.

In addition to being positive for virus isolation, five of the six control animals were also positive for anti-E^rns^ antibodies, an antigen not included in the vaccine. Only one of the control animals challenged with isolate PR-11/10-3 was negative for anti-E^rns^ antibodies. In the three other animals, the test could not be run because they died early with clinical signs of CSF. These findings indicate that the anti-E^rns^ antibody test can be helpful in discriminating between vaccinated and infected animals (DIVA). 

In a previous study, only three out of ten five-day-old piglets infected with the same isolate PR-11/10-3 seroconverted to E^rns^ [20]. Our experiment was conducted in older pigs, nine weeks old, and this difference in age could account for the divergent results found in the frequency of E^rns^ seroconversion against this isolate. Previous results for other groups have suggested that the virulence of the infecting viral strain exerts a marked influence on the frequency and timing of E^rns^ seroconversion [21]. The use of E^rns^ ELISAs to discriminate between vaccinated and infected pigs has been controversial. Early evaluations concluded that these ELISAs were less sensitive than conventional CSF E2 antibody ELISAs [22,23]. However, although the sensitivity of these ELISAs is not optimal, they can still be useful when applied on a herd basis to a given number of animals [23].

Animals vaccinated with Porvac^®^ seroconverted from the first immunization and developed elevated NAb titers following the booster. None of these vaccinated animals showed clinical signs of CSF after challenge with the three Cuban isolates. Virus could not be isolated from blood or organ samples, and the animals did not develop anti-E^rns^ antibodies. These results strongly suggest that Porvac^®^ is able to protect pigs from infection with these three field isolates, which have low, medium or medium–high virulence and some amino acid changes in the E2 protein relative to the vaccine strain. In this experiment, it was not possible to measure the viral load of pigs with the real-time RT-PCR assay, which has higher sensitivity to detect virus remnants in tissues or blood. However, given its extreme sensitivity, RT-PCR can yield positive results, with high CT values, in the absence of replicative virus; therefore, virus isolation is the gold-standard technique to demonstrate the existence of replicative CSFV in sera and organs. In the particular case of isolate PR-11/10-3, further studies with a larger number of animals and controls and a longer follow-up time are required to provide a more conclusive answer on the protection conferred by Porvac^®^ against this very low-virulence isolate. It would be also of interest to test if animals infected very early in life with this isolate could mount an immune response after Porvac^®^ vaccination, after a chronic silent infection has been established.

The ability of Porvac^®^ to protect against low-, medium-, and high-virulence autochthonous CFSV isolates supports its use in a vaccination campaign for the control and gradual elimination of CSF in Cuba.

## 5. Conclusions

1. The unvaccinated control animals developed clinical disease according to the virulence of the different isolates with which they were challenged. PR-11/10-3 and Holguín_2009 showed very mild and moderate disease, respectively, while PR-2016 showed a clinical picture compatible with higher virulence.
2. Unlike the controls, the vaccinated animals did not show clinical signs of CSF or circulating virus after challenge, so it is possible to affirm that the immune response induced by Porvac^®^ protected against challenge with the three Cuban isolates of CSF virus.
3. The detection of anti-E^rns^ antibodies after challenge allowed the pigs vaccinated with Porvac^®^, which were all negative, to be distinguished from the infected controls (83.3% positivity).

## Figures and Tables

**Table 1:** Anti-E^rns^ antibodies in blood after challenge.

Group	Animal	14 dpc	21 dpc	28 dpc
1	669	-	-	-
	670	-	-	-
	671	-	-	-
	673	-	-	-
	674	-	-	-
2	675	-	-	-
	676	-	-	-
	677	-	-	-
	678	-	-	-
	679	-	-	-
3	680	-	-	-
	681	-	-	-
	682	-	-	-
	683	-	-	-
	684	-	-	-
4	689	-	-	-
	690	-	-	+
	691	ND	-	+
5	692	ND	+	+
	694	ND	+	+
	695	†	†	†
6	696	ND	†	†
	697	ND	†	†
	698	ND	+	+

dpc: days post-challenge; ND: not done; †: death of pig, +: positive; -: negative. Groups 1, 2, and 3: pigs vaccinated with Porvac^®^; groups 4, 5 and 6: non-vaccinated pigs. Groups 1 and 4: challenged with PR-11/10–3; groups 2 and 5: challenged with Holguín_2009; groups 3 and 6: challenged with PR-2016.

**Table 2:** Viral isolation from blood and organs after challenge.

		Blood				Organs		
Groups	Animal	7 dpc	14 dpc	21 dpc	28 dpc	Tonsils	Spleen	Lungs
1	669	-	-	-	-	-	-	-
	670	-	-	-	-	-	-	-
	671	-	-	-	-	-	-	-
	673	-	-	-	-	-	-	-
	674	-	-	-	-	-	-	-
2	675	-	-	-	-	-	-	-
	676	-	-	-	-	-	-	-
	677	-	-	-	-	-	-	-
	678	-	-	-	-	-	-	-
	679	-	-	-	-	-	-	-
3	680	-	-	-	-	-	-	-
	681	-	-	-	-	-	-	-
	682	-	-	-	-	-	-	-
	683	-	-	-	-	-	-	-
	684	-	-	-	-	-	-	-
4	689	-	-	-	-	-	-	+
	690	-	-	-	-	-	-	+
	691	-	-	-	-	-	-	+
5	692	+	-	-	-	-	-	-
	694	-	-	-	-	-	-	+
	695	-	†	-	†	-	-	+
6	696	+	+	†	†	+	+	+
	697	+	+	†	†	+	+	+
	698	+	+	-	-	-	+	+

dpc: days post-challenge; †: death of pig; +: positive; -: negative. Groups 1, 2, and 3: pigs vaccinated with Porvac^®^; groups 4, 5, and 6: non-vaccinated pigs. Groups 1 and 4: challenged with PR-11/10–3; groups 2 and 5: challenged with Holguín_2009; groups 3 and 6: challenged with PR-2016.

## Data Availability

Data presented in this study are available on request from the corresponding author.

## References

[1] Ganges L., Crooke H.R., Bohórquez J.A., Postel A., Sakoda Y., Becher P., Ruggli N. (2020). Classical swine fever virus: The past, present and future. Virus Res..

[2] Brown V.R., Miller R.S., McKee S.C., Ernst K.H., Didero N.M., Maison R.M., Grady M.J., Shwiff S.A. (2021). Risks of introduction and economic consequences associated with african swine fever, classical swine fever and foot-and-mouth disease: A review of the literature. Transbound. Emerg. Dis..

[3] Hu D., Lv L., Gu J., Chen T., Xiao Y., Liu S. (2016). Genetic diversity and positive selection analysis of classical swine fever virus envelope protein gene e2 in east china under c-strain vaccination. Front. Microbiol..

[4] Ji W., Niu D.D., Si H.L., Ding N.Z., He C.Q. (2014). Vaccination influences the evolution of classical swine fever virus. Infect. Genet. Evol. J. Mol. Epidemiol. Evol. Genet. Infect. Dis..

[5] Perez L.J., Diaz de Arce H., Perera C.L., Rosell R., Frias M.T., Percedo M.I., Tarradas J., Dominguez P., Nunez J.I., Ganges L. (2012). Positive selection pressure on the b/c domains of the e2-gene of classical swine fever virus in endemic areas under c-strain vaccination. Infect. Genet. Evol. J. Mol. Epidemiol. Evol. Genet. Infect. Dis..

[6] Luo Y., Ji S., Lei J.-L., Xiang G.-T., Liu Y., Gao Y., Meng X.-Y., Zheng G., Zhang E.-Y., Wang Y. (2017). Efficacy evaluation of the c-strain-based vaccines against the subgenotype 2.1 d classical swine fever virus emerging in china. Vet. Microbiol..

[7] Frías-Lepoureau M.T., Greiser-Wilke I., Morilla A., Hernandez P., Yoon J.K., Zimmerman J. (2002). An update on classical swine fever (csf) virus molecular epidemiology. Trends in Emerging Viral Infections of Swine.

[8] Diaz de Arce H., Nunez J.I., Ganges L., Barreras M., Teresa Frias M., Sobrino F. (1999). Molecular epidemiology of classical swine fever in cuba. Virus Res..

[9] Díaz de Arce H., Ganges L., Barrera M., Naranjo D., Sobrino F., Frias M.T., Nunez J.I. (2005). Origin and evolution of viruses causing classical swine fever in cuba. Virus Res..

[10] Van Rijn P., Miedema G., Wensvoort G., Van Gennip H., Moormann R. (1994). Antigenic structure of envelope glycoprotein e1 of hog cholera virus. J. Virol..

[11] Van Rijn P.d., Van Gennip H., De Meijer E., Moormann R. (1993). Epitope mapping of envelope glycoprotein e1 of hog cholera virus strain brescia. J. Gen. Virol..

[12] Sordo-Puga Y., Suárez-Pedroso M., Naranjo-Valdéz P., Pérez-Pérez D., Santana-Rodríguez E., Sardinas-Gonzalez T., Mendez-Orta M.K., Duarte-Cano C.A., Estrada-Garcia M.P., Rodríguez-Moltó M.P. (2021). Porvac^®^ subunit vaccine e2-cd154 induces remarkable rapid protection against classical swine fever virus. Vaccines.

[13] Coronado L., Rios L., Frías M.T., Amarán L., Naranjo P., Percedo M.I., Perera C.L., Prieto F., Fonseca-Rodriguez O., Perez L.J. (2019). Positive selection pressure on e2 protein of classical swine fever virus drives variations in virulence, pathogenesis and antigenicity: Implication for epidemiological surveillance in endemic areas. Transbound. Emerg. Dis..

[14] Coronado L., Liniger M., Muñoz-González S., Postel A., Pérez L.J., Pérez-Simó M., Perera C.L., Frías-Lepoureau M.T., Rosell R., Grundhoff A. (2017). Novel poly-uridine insertion in the 3′ utr and e2 amino acid substitutions in a low virulent classical swine fever virus. Vet. Microbiol..

[15] Coronado L., Bohórquez J.A., Muñoz-González S., Perez L.J., Rosell R., Fonseca O., Delgado L., Perera C.L., Frías M.T., Ganges L. (2019). Investigation of chronic and persistent classical swine fever infections under field conditions and their impact on vaccine efficacy. BMC Vet. Res..

[16] Suárez M., Sordo Y., Prieto Y., Rodríguez M.P., Méndez L., Rodríguez E.M., Rodríguez-Mallon A., Lorenzo E., Santana E., González N. (2017). A single dose of the novel chimeric subunit vaccine e2-cd154 confers early full protection against classical swine fever virus. Vaccine.

[17] EMA (2000). VICH G09. Guideline Ongood Clinical Practices. www.ema.europa.eu/en/documents/scientific-guideline/vich-gl9-good-clinical-practices-step-7_en.pdf.

[18] Mittelholzer C., Moser C., Tratschin J.D., Hofmann M.A. (2000). Analysis of classical swine fever virus replication kinetics allows differentiation of highly virulent from avirulent strains. Vet. Microbiol..

[19] WOAH Manual of Diagnostic Tests and Vaccines for Terrestrial Animals, Chapter 3.9.3. Classical Swine Fever (Infection with Classical Swine Fever Virus). https://www.woah.org/fileadmin/Home/eng/Health_standards/tahm/3.09.03_CSF.pdf.

[20] Wang M., Liniger M., Muñoz-González S., Bohórquez J.A., Hinojosa Y., Gerber M., López-Soria S., Rosell R., Ruggli N., Ganges L. (2020). A polyuridine insertion in the 3′ untranslated region of classical swine fever virus activates immunity and reduces viral virulence in piglets. J. Virol..

[21] Moormann R.J., Bouma A., Kramps J.A., Terpstra C., De Smit H.J. (2000). Development of a classical swine fever subunit marker vaccine and companion diagnostic test. Vet. Microbiol..

[22] Floegel-Niesmann G. (2001). Classical swine fever (csf) marker vaccine: Trial iii. Evaluation of discriminatory elisas. Vet. Microbiol..

[23] Pannhorst K., Fröhlich A., Staubach C., Meyer D., Blome S., Becher P. (2015). Evaluation of an erns-based enzyme-linked immunosorbent assay to distinguish classical swine fever virus–infected pigs from pigs vaccinated with cp7_e2alf. J. Vet. Diagn. Investig..

